# A Case of Complete Response to Letrozol Treatment in a Postmenopausal Woman With Breast Cancer Who has Progressed After Multiple Lines of Chemotherapy

**DOI:** 10.4021/wjon628w

**Published:** 2013-03-06

**Authors:** Deniz Tural, Emre Akar, Sait Sager, Ozcan Yildiz, Mustafa Ozguroglu

**Affiliations:** a Department of Internal Medicine, Istanbul University, Cerrahpasa Medical Faculty, Division of Medical Oncology, Istanbul, Turkey; b Istanbul University, Cerrahpasa Medical Faculty, Istanbul, Turkey; c Department of Nuclear Medicine, Istanbul University, Cerrahpasa Medical Faculty, Istanbul, Turkey

**Keywords:** Metastatic breast cancer, Letrozol, Complete response

## Abstract

Approximately 60% of all breast cancers are endocrine dependent. Postmenopausal patients who have positive hormone receptor status are eligible for aromatase inhibitor treatment. Letrozole is a potent, selective, non-steroidal, third-generation aromatase inhibitor which reduces oestrogen biosynthesis approximately 99% at the dose of 2.5 mg/day. We report a 54-years-old female patient diagnosed with grade 2 invasive ductal carcinoma of the breast. She received adjuvant chemotherapy, followed by 5 years of tamoxifen. After 8 years, recurrence appeared in lung, supraclavicular lymph nodes and brain. She had many cycles of cytotoxic chemotherapeutic agents, trastuzumab and lapatinib previously. After the progression (lung and brain), palliative therapy was thought due to very poor performance status of the patient. (ECOG: 3) Letrozole was added in the treatment and we obtained near-complete remission from her lung and brain metastasis with 2.5 mg/day dose of letrozole. This study might support successfully use of aromatase inhibitors in patients who has been previously treated with multiple lines of chemotherapy and had still progressive disease.

## Introduction

Breast cancer is the most frequently diagnosed cancer and the leading cause of cancer death in females [1]. In addition, approximately 60% of all breast cancers are endocrine dependent. Endocrine therapies are targeted at oestrogen that cancer cells need in order to proliferate. Anti-oestrogen drugs such as tamoxifen can block the oestrogen receptor in nucleus of the responding cells. In postmenopausal women, oestrogens are converted from androgens by the aromatase enzyme that is the target of aromatase inhibitors [2]. Patients who have positive hormone receptor status are eligible for aromatase inhibitor treatment. Letrozole is a potent, selective, non-steroidal, third-generation aromatase inhibitor that reduces oestrogen biosynthesis approximately 99% at the dose of 2.5 mg/day [3].

Indeed, the treatment of breast cancer in postmenopausal women whose disease is progressing despite having more cycles of cytotoxic and hormonal anti-cancer therapy, presents a number of challenges. Moreover, poor performance status (ECOG ≥ 2) is encountered often in these patients. Our knowledge and experience need to increase about choosing the next drug and its success in metastatic breast cancer patients that need palliative treatment.

We report here on a patient with metastatic breast cancer who obtained near-complete response with letrozole treatment after progressive disease with many cycles of cytotoxic chemotherapy and ECOG: 3 performance status.

## Case Report

**Figure 1:**
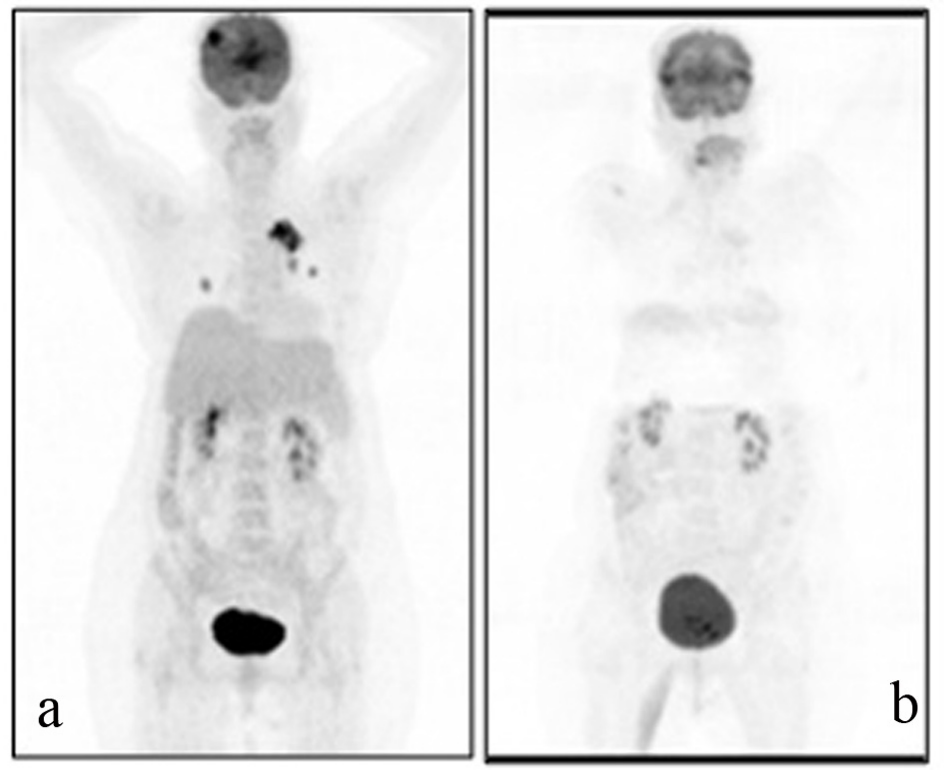
A 69-year-old female patient with a history of breast cancer underwent FDG PET/CT imaging before and after the therapy. For the first PET/CT imaging patient was intravenously injected 592 MBq (16 mCi) F-18 FDG after 6 hours of fasting period. Then one hour of waiting time in a silent room patient was imaged using an integrated PET/CT camera, which was consisted of a 6-slice CT gantry integrated on a LSO based full-ring PET scanner (Siements Biograph 6, IL, USA). Anterior-posterior maximum intensity projection (MIP) PET image (a) sowed intense hypermetabolic multiple nodular lesions in the right lung superior lobe with a maximum standard uptake value (SUV max) of 14.0 and left hilar hypermetabolic lymph node with a SUVmax of 6.4. In cranial slices intense hypermetabolic lesion in the right frontal lobe with a SUVmax of 13.5 was seen. Six months after the first PET/CT imaging this patient referred to Nuclear Medicine department for another PET/CT imaging after the therapy to evaluate the therapy response. Patient wan injected 418.1 MBq (11.3 mCi) F-18 FDG for imaging. Anterior and posterior MIP image (b) showed significant regression in the cranial, lung and left hilar lesions.

A 54-years-old female patient was diagnosed with grade 2 invasive ductal carcinoma of the breast in 1997. Modified radical mastectomy was performed to the patient who has spread disease to sentinel lymph nodes. Post-operative pathological examination revealed stage II breast cancer with two out of 14 lymph nodes positive (pN1), ER 3+, PR 3+, and CERB-B2 3+. Adjuvant radiotheraphy and chemotheraphy with 4 cycles anthracycline cyclophosphamide and 4 cycles docetaxel was performed and 5-year adjuvant treatment of tamoxifene was commenced. In 2005, recurrence appeared in lung and supraclavicular lymph nodes and she was treated for 6 cycles with trastuzumab and vinorelbine. Trastuzumab and vinorelbine were continued for two and a half years and there was no evidence of progression in this period. Lapatinib and capecitabine were commenced after progression that was observed on CT scan in lung lesions and 22 cycles of this treatment were performed. F-18 Fluorodeoxyglucose Positron Emmition Tomography (PET-CT), performed in order to confirm the extent of metastases of the patient whose CT imaging revealed lung lesions, revealed metastatic lesion in her lungs. Cranial MRI was performed to the patient who complained from headache and whose PET-CT imaging revealed cranial metastasis. Cranial radiotherapy (3 gy/day, total 30 gy) was performed to the patient for her frontal lobe lesions. Given her poor performance status (ECOG: 3) and hormone receptor positivity of primary tumor, palliative therapy and letrozole (2.5 mg/day) was started after the radiotherapy. Control PET-CT was performed after six months (Fig. 1a, b) and revealed near-complete response as a result that was obtained with the letrozole treatment.

## Discussion

The success of aromatase inhibitors in postmenopausal women with breast cancer increased the importance of these anti-hormonal agents. The new generations of aromatase inhibitors are letrozole, anastrozole and exemestane. These agents were directly compared with tamoxifen and letrozole is the only aromatase inhibitor to have shown superiority over tamoxifen as first line treatment in postmenopausal patients [4, 5]. There is a previous publication regarding the efficient use of anastrozole in patients who have advanced breast cancer and treated with prior multiple cytotoxic chemotherapies and endocrine therapy [6]. However, there is lack of data about successful use of letrozole in these patients. Letrozole is indicated in the treatment of postmenopausal patients with HR + breast cancer in both the early and metastatic settings. In male breast cancer, there are two publications about successful use of letrozole and in one of these patients, complete response was obtained with letrozole after failure of tamoxifen [7, 8].

Our patient had many cycles of cytotoxic chemotherapeutic agents previously (anthracycline cyclophosphamide, docetaxel, doxorubicin) and tamoxifen as endocrine therapy. Furthermore, trastuzumab and lapatinib was given to the patient with the aim of to receive a response. After the progression despite all these treatments, palliative therapy was thought due to very poor performance status of the patient. Letrozole was added in the treatment with expecting of benefit, taking into consideration that the patient has not received any aromatase inhibitor before. We obtained near-complete remission from her lung metastasis with 2.5 mg/day dose of letrozole. However, due to cranial radiotherapy, reducing of the brain metastasis may not be connected entirely with the use of letrozole.

Given all this information, letrozole might be appropriate for treatment of hormone receptor positive metastatic breast cancer that other anti-cancer agents failed to respond well. On the other hand, this report raises the question of the place of letrozole as addition of palliative therapy in ECOG > 2 patients. We demonstrate successful use of letrozole in this situation but only in one patient. Further and prospective clinical studies are required to help understanding the place of aromatase inhibitors in condition of progression after multiple prior anti-cancer agents and poor performance status.

## Declaration

All authors participate this study equally.
